# Measuring the tail of the dog that doesn't bark in the night: the case of the national evaluation of Choose Life (the national strategy and action plan to prevent suicide in Scotland)

**DOI:** 10.1186/1471-2458-7-146

**Published:** 2007-07-06

**Authors:** Mhairi Mackenzie, Avril Blamey, Emma Halliday, Margaret Maxwell, Allyson McCollam, David McDaid, Joanne MacLean, Amy Woodhouse, Stephen Platt

**Affiliations:** 1 Department of Urban Studies, University of Glasgow, Glasgow, Scotland, UK; 2 NHS Health Scotland, Edinburgh, UK; 3 Department of General Practice, University of Edinburgh, Edinburgh, UK; 4 Scottish Development Centre for Mental Health, Edinburgh, UK; 5 LSE Health and Social Care & European Observatory on Health Systems and Policies, London School of Economics and Political Science, London, UK; 6 Research Unit in Health, Behaviour and Change, School of Clinical Sciences and Community Health, University of Edinburgh, Edinburgh, UK

## Abstract

**Background:**

Learning about the impact of public health policy presents significant challenges for evaluators. These include the nebulous and organic nature of interventions ensuing from policy directives, the tension between long-term goals and short-term interventions, the appropriateness of establishing control groups, and the problems of providing an economic perspective. An example of contemporary policy that has recently been subject to evaluation is the first phase of the innovative Scottish strategy for suicide prevention (*Choose Life*).

**Discussion and summary:**

This paper discusses how challenges, such as those above, were made manifest within this programme. After a brief summary of the overarching approach taken to evaluating the first phase of *Choose Life*, this paper then offers a set of recommendations for policymakers and evaluators on how learning from a second phase might be augmented. These recommendations are likely to have general resonance across a range of policy evaluations as they move from early planning and implementation to more mature phases.

## Background

In recent years two interrelated sets of arguments about the evaluation of public health policy have become commonplace. The first laments the lack of a robust evidence base for developing policy, and charges evaluators to do better in producing such knowledge. This, it is argued, should be brought about by the more frequent application of gold-standard approaches to complex interventions [1,2] and through adopting 'a more integrated approach to evaluation and implementation, using the most robust and sound methodologies, and taking advantage of "natural experiments"' (p.40) [3]. This is a view endorsed by the 2004 Wanless report on improving population health [4].

The second argues that both evaluators and policymakers need to be more explicit about the kinds of knowledge that they want to generate from the study of such programmes, and the place of this evidence in subsequent policymaking [5-9].

In this paper, within the context of these debates, we exemplify the challenges inherent in evaluating *Choose Life*: *The National Strategy and Action Plan to Prevent Suicide in Scotland*. We then briefly summarise the overarching approach taken to evaluating the first phase of *Choose Life*. Finally, we set out recommendations for framing subsequent evaluation of the initiative as it enters its more mature second phase. These recommendations have implications for policymakers and evaluators within public health and in wider policy domains.

*Choose Life *was launched in 2002 as a major strand within the National Programme for Improving Mental Health and Well-being in Scotland [10]. As with much contemporary policy emanating from the Scottish Executive, the National Programme was charged with tackling health inequalities within a wider health improvement agenda [11]. The overall aim of Choose Life was to reduce suicide by 20% over a ten year period through the achievement of objectives such as improved early prevention and crisis response, engagement with the media and, adoption of an evidence-based approach. More detail on the guiding principles of *Choose Life *and its objectives and priority groups is presented in table 1 below.

**Table 1:** Choose Life: *Principles, Objectives and Priority Groups*

***Guiding principles***
• Shared responsibility (across Scottish Executive departments, sectors, agencies and organisational boundaries)
• Effective leadership (nationally and locally
• Taking a person-centred approach (recognising variation in individuals' experiences, often associated with key life stages)
• Focus on priority approach (without losing sight of the broader needs of society as a whole)
• Continuous quality improvement (drawing on, and developing, better information and evidence of what works)
***Main objectives***
• Early prevention and intervention
• Responding to immediate crisis
• Longer-term work to provide hope and support recovery
• Coping with suicidal behaviour and completed suicide
• Promoting greater public awareness and encouraging people to seek help early
• Supporting the media
• Knowing what works

***Priority groups***
• Children (especially looked after children)
• Young people (especially young men)
• People with mental health problems (particularly service users and people with severe mental illness)
• People who attempt suicide
• People affected by the aftermath of suicidal behaviour
• People who abuse substances
• People in prison
• People who are recently bereaved
• People who have recently lost employment or who have been unemployed for a period of time
• People in isolated or rural communities
• People who are homeless

The *Choose Life *plan was to be implemented in two phases from 2003–2013. In phase one (2003–2006) £9 m was allocated to local partnerships across each local authority area in Scotland, to be used for suicide prevention work, whilst £3 m was set aside for national activities in support of the plan. Local areas were, in addition, positively encouraged to generate additional resources for *Choose Life *activities. A further £8.4 m was set aside for the first two years of the second phase (2006–2008). A more detailed description of the structure of the initiative is provided by Platt and colleagues [12].

An intention to evaluate phase one of *Choose Life *was signalled in the national strategy and action plan [10] and, following a competitive tendering process, a consortium of researchers at the Universities of Edinburgh and Glasgow, the London School of Economics and the Scottish Development Centre for Mental Health was awarded a two year contract commencing in mid-2004.

## Discussion

A recent comparative review of suicide prevention strategies across the globe, carried out as part of the commissioned *Choose Life *evaluation, highlighted that the majority of government-funded efforts to generate learning about effective approaches have focused on evaluating specific interventions rather than on national strategies *per se *[12]. For example, Mann and colleagues, in their review of suicide prevention strategies, look at the impact of individual components of strategies rather than at the effect of an overall programme of activity [13]. Whilst Australia commissioned a summative evaluation of its national youth suicide prevention strategy, Scotland was unique in its investment in a national level formative evaluation although other countries are now following suit. Although this provided a welcome opportunity to learn how whole systems create the context for effective suicide prevention strategies, it raised an array of challenges for would-be evaluators.

In this section we consider four specific issues that needed to be addressed in the evaluation of the first phase of *Choose Life*: how to learn from a complex initiative with multiple stakeholders and outcomes; understanding secular trends in reaching evaluative judgements (effectiveness); attempting to provide an economic perspective on the worth of a national programme (cost-effectiveness); and integrating data arising from a range of methodological approaches. This list, while not exhaustive, represented the most significant challenges for the evaluation team.

### The nature of complexity in policy initiatives

Current approaches to making and implementing policy add to programme complexity in a number of ways. Here we briefly outline seven elements of this complexity and illustrate in Table 2 below the ways in which these were made manifest in *Choose Life*.

**Table 2:** Challenges of Complexity

**Nature of the challenge**	**Its manifestation in *Choose Life***
i. The multiple purposes that evaluation is anticipated to serve	The national evaluation team had objectives in relation to: monitoring progress towards implementation of programme milestones; examining the extent to which effective practice was being generated; assessing the development of sustainable infrastructure; providing summative as well as formative feedback.
ii. Multiple outcomes of the programme	The programme had seven multi-faceted objectives. These were:
	1) Early prevention and intervention: providing earlier intervention and support to prevent problems and reduce the risks that might lead to suicidal behaviour;
	2) Responding to immediate crisis: providing support and services to people at risk and people in crisis, to provide an immediate crisis response and to help reduce the severity of any immediate problem;
	3) Longer-term work to provide hope and support recovery: providing on-going support and services to enable people to recover and deal with the issues that may be contributing to their suicidal behaviour;
	4) Coping with suicidal behaviour and completed suicide: providing effective support to those who are affected by suicidal behaviour or a completed suicide;
	5) Promoting greater public awareness and encouraging people to seek help early: ensuring greater public awareness of positive mental health and well-being, suicidal behaviour, potential problems and risks amongst all age groups, and encouraging people to seek help early;
	6) Supporting the media: ensuring that any depiction or reporting by any section of the media of a completed suicide or suicidal behaviour is undertaken sensitively and appropriately and with due respect for confidentiality; and,
	7) Knowing what works: improving the quality, collection and availability of information on issues relating to suicide and suicidal behaviour and on effective interventions to ensure the better design and implementation of responses and services and use of resources.
iii. Horizontal complexity	The local authority areas received their funding through the Community Planning Partnership mechanism. These statutory planning groups had representation from across public sector organisations (such as the NHS, local government and education) and the voluntary sector. The national level also required partnerships to be made and sustained between a wide range of organisations.
iv. Vertical complexity	Impacts were anticipated among individuals at risk, their families, the organisations with which they engage, and at a structural level.
v. The lack of opportunity to establish control/comparison groups	All local authority areas in Scotland were provided with funding to take forward *Choose Life*. It was not possible, therefore, to identify areas within the country that could act as 'controls'.
vi. Context as integral to a programme intervention	Local areas were given flexibility in how they organised the local manifestations of *Choose Life*. For example, they were able to identify their own local priority groups in addition to those identified by the Scottish Executive, determine how their funding would be utilised, and develop their own management arrangements for the programme of work undertaken.
vii. Long-term goals	To reduce the rate of suicide in Scotland by 20% by 2013

First, there is a growing recognition that there has been an expansion in the number and types of roles that evaluators are expected to play [14-16]. In particular, policy evaluators are now expected to provide developmental support to programmes as well as to make judgements of impact, and to provide formative as well as summative feedback. Whilst the evaluation of the first phase of *Choose Life *was explicitly focused on the process of implementation, there were, nonetheless, requests from funders for the evaluation team to answer questions of effectiveness. Negotiating, and remaining focused on, the *purpose *of an evaluation is, therefore, a key challenge; such purpose will, in addition, change as the evaluation moves from a developmental to a theory-testing stage.

Second, national programmes involving a wide range of stakeholders inevitably involve multiple outcomes [17]. *Choose Life *is a useful example of this. Although the national strategy set itself one long-term target (to reduce the suicide rate in Scotland by 20% between 2002 and 2013), the local areas were given seven key objectives and were expected to meet 12 milestones (progress towards these measures is detailed by Platt and colleagues [12]. Multiple outcomes do not simply muddy the waters for evaluators through increasing the range of data to be collected. A feature of complex initiatives is that there is a general lack of clarity about when change across and between outcomes is anticipated because of the unknown synergies that may occur [18]. The evaluator, therefore, can struggle to identify the most appropriate point at which to look for outcomes and the order in which they will appear. Furthermore stakeholders' outcomes can be incompatible and disagreements can occur regarding the primary focus of an impact assessment.

Third, as described by Connell and Kubisch [17], strategic approaches to tackling health and social problems exhibit 'horizontal complexity'. This means that a range of different organisations, often with mismatched agendas, have a stake in the outcomes (or funding) of an initiative. This may be particularly problematic where funding is disbursed locally to partnership bodies (Community Planning Partnerships in the case of *Choose Life*) whose member organisations have different approaches to mental health promotion and to how evidence is gathered and interpreted. In addition, the *Choose Life *programme manifested horizontal complexity at a national level, with its National Implementation Support Team charged with working in partnership with other bodies at a Scotland-wide level. Once again, experience of evaluating such initiatives has demonstrated that the evaluator has a tricky balance to maintain in satisfying the requirements of different stakeholders.

Fourth, complexity is further increased in strategies that aim to impact on organisations, specific professionals and practices, communities and individuals ('vertical complexity'). Potential outcomes can start to multiply exponentially [17]. Evaluators cannot and should not measure everything: they need to develop a purposive strategy for determining what will be assessed and maintain a focus on this throughout the course of an evaluation.

Fifth, evaluators who seek to use controlled research designs to demonstrate that observed changes can be attributed to the implementation of the programme may find that complex initiatives are ill-suited to such approaches. Where programmes are delivered in a small number of pilot areas, the thrust of policy momentum can result in programme diffusion to what might have been regarded as appropriate control sites [7,8]. This is clearly exacerbated in strategies where no pilot work is undertaken and an initiative is launched across an entire country, as with *Choose Life*. Some commentators have argued that, with minor design modifications, the practical problems of carrying out randomised controlled trials can be overcome [19]. The political realities of strategy development and implementation within the UK, however, throw this into question. A randomised design is also compromised by the lack of comparability of interventions and their implementation processes between intervention areas.

A sixth ingredient of complexity is that programmes are shaped by the contexts in which they are located. The body of literature emerging from a theory-based approach to evaluation argues that it is inappropriate and simplistic to ask *whether *an intervention works, since an intervention is made up of a myriad of interacting components, and because some elements will work for particular groups of individuals in specific contexts and circumstances [20-22]. Each of the local areas charged with implementing *Choose Life *has its own organisational history and was given freedom to deliver the programme in locally determined ways. Understanding context, therefore, becomes a more important evaluation task than ironing it out through a controlled design. Nevertheless, understanding the contribution made by a programme to observed change remains a concern for evaluators. We expand on this in the following section.

Finally, complex health and social programmes are launched to tackle the most intractable problems. Their long-term goals, therefore, are ambitious and, more importantly, lie beyond the timescales of the interventions set up to deliver them and the evaluations designed to measure them. This challenges evaluators to select plausible, robust and short-term intermediate or proxy measures of the more distal intended outcome(s). In the metaphor of our title, the long term outcome of interest is the absence of suicide events (the dog that doesn't bark in the night). Whilst we cannot realistically capture this within the life of an evaluation, the evaluator's goal is to ensure that process measures are appropriate proxies for this ultimate outcome (in other words, to make a case for the measurement of the tail of the dog as a predictor of its future barking behaviour). In suicide prevention evaluations the quest for appropriate proxies is made difficult by the deficiencies of the existing evidence base. There remains a significant leap in the dark, for example, from the provision of mental health awareness programmes in secondary school to the prevention of suicide attempts in the community at some future date. Likewise, there is debate about the value of proxies such as hospital-treated self-harm as a marker for later suicide [23].

### Secular trends

As discussed above, demonstrating that a programme has led to a change in outcome is not in itself enough to convince observers that the intervention has played a causal role. This is true in particular when trends in the outcome of interest are on a downward slope. For example, the rates for suicides and undetermined deaths for men in Scotland rose to a peak in 1992 (34.2/100,000 population aged 15+ years), remained at (or just below) this high level until 2002 (34.1/100,000), after which there has been a decline to 27.1/100,000 in 2005 (lowest rate since 1991) [12]. The decline since 2002 may be attributable in part to *Choose Life *activities. However, the massive reduction in male suicide and undetermined deaths between 2002 (n = 673, rate = 34.1/100,000) and 2003 (n = 577, rate = 29.1/100,000) occurred when *Choose Life *had only been very partially implemented and is instead probably due, in large part, to the influence of other factors (e.g. legislation restricting paracetamol sales [23-25]). In the absence of control data there is, therefore, a significant challenge in interpreting trends over time.

This is exacerbated by the relative rarity of suicide as an event. It becomes virtually impossible for evaluators to propose (dis)confirmable explanations for observed changes in trends over short time periods (in this instance, two years) and at a small area level. Teasing out the possible impact of different strategic approaches within the umbrella of *Choose Life *on rates of suicide is equally problematic. Once again this points to the need for plausible short-term indicators of progress but without evidence of strong indicators of suicidal behaviour this remains difficult.

### Undertaking an economic analysis

As debates about scarcity of resources within the NHS grow, there is a parallel increase in policy interest in addressing the cost-effectiveness of differing approaches to health improvement, yet economic evaluations of complex public health interventions remain relatively rare [26]. Sefton [27] has argued, that bringing health economic perspectives from the field of narrowly defined technology assessment into the domain of complex policy evaluation raises both practical and epistemological difficulties. First and foremost, approaches to assessing cost-effectiveness rely on the existence of robust measures of both costs and benefits. At the level of policy and public health interventions, however, these types of data are woefully limited and the problems that beset randomised controlled trials also undermine the use of standard health economic techniques [28]. In addition, the economic notion of time preference has meant that short term outcomes, more likely to be seen in health care interventions, are usually deemed to have greater economic benefits than health promoting interventions that may take several years to achieve change. [30,31] Second, paradigmatic conflicts between those working in the field of traditional outcome studies and those dealing with the complexities of messy policy evaluation can result in stereotypical and blinkered views of the contribution to be made by different disciplines and research approaches. For example, McDaid and colleagues argue that, within the field of suicide prevention, an economist's focus on a single outcome, such as the rate of suicide or the number of life years saved, may be viewed as inappropriately reductionist by those interested in capturing the complexity of synergistic approaches to mental health promotion [28]. Conversely, a focus on process and organisational change to the detriment of measurable outcomes may be viewed by economists as a failure to pursue an intervention's bottom line. As Sefton argues, making these 'ends meet' requires not only that evaluation teams are multidisciplinary in name but also that they practise in an integrative fashion [27].

### Integrating findings

Achieving an integrated approach to evaluation is much less straightforward than it sounds. Accepting the need to address questions of process and outcome and designing an evaluation that will seek to gather data which are appropriate to both can still result in 'silo evaluations', with the final report summarising results in separate sections. In their evaluation of the Total Purchasing Pilot in primary care, Evans and colleagues illustrate this through their description of how, in the absence of an *a priori *theoretical framework, members of a multidisciplinary team were left to undertaken their own sub-components of a multi-method evaluation [32]. Even explicit and pre-emptive attempts to be integrative, such as the use of theory-based approaches to evaluation, can struggle to combine data from different sources [7].

### Our approach

Although aware of the challenges that have beset policy evaluations in recent years when we embarked on our evaluation of the first phase of Choose Life, we nevertheless struggled to find satisfactory solutions through our evaluation approach and practice. Here we summarise the main aims of the national evaluation of *Choose Life *as set out within the research brief and the general evaluation framework that we used.

At its most general level the purpose of the national evaluation was to 'assess ... infrastructure and early impacts' of Choose Life and to 'set the template for the next phase' of the strategy [10]. Its aims are detailed in table 3 below:

**Table 3:** National Evaluation of Choose Life – Main Aims

1. Establish and apply measures to assess whether a sustainable infrastructure is being developed nationally and locally to support the *Choose Life *strategy in achieving its objectives
2. Measure and review progress towards implementation of the 27 milestones identified in the Choose Life strategy and action plan and set findings in context, national and internationally
3. Examine whether and how Choose Life is stimulating effective forms of practice (nationally and in individual local areas)
4. On the basis of findings, and in consultation with the Scottish Executive and the Research Advisory Group steering the evaluation, provide detailed and staged recommendations to guide the next phase of the action plan to achieve a 20 per cent reduction in suicides in Scotland by 2013, and the targeting of any funding available to support the next phase

Our overarching evaluation framework derived from Theories of Change [20,22]. This approach, designed specifically for the evaluation of complex, multiple outcome and multi-partner programmes, aims to study cumulatively the links between the purposive activities undertaken to tackle significant social problems, their short-term outcomes and processes, and longer-term goals. It seeks to address questions about which components of an overall programme work for which individuals in which sets of circumstances. In this regard it is well suited to the evaluation of a national strategy that is expected to operate differently in different local contexts, since it aims to capture why and how local variations occur. It also explicitly recognises that different stakeholders within an intervention will have potentially competing expectations of a programme and seeks to deal with this by encouraging a collaborative approach to agreeing a theory of change and prioritising key indicators and outcomes for tracking progress.

Whilst the approach is not without its practical and conceptual difficulties [33-35], it does nonetheless aim to provide an integrative framework for data generation and interpretation. For example, using the approach allows questions of effectiveness and cost-effectiveness to be embedded in a chain of events linking complex organisational processes, monitoring data and proxy measures of programme success. Its main difficulties include its time-consuming nature, the challenges of eliciting detailed and well-specified theories from stakeholders, and (of particular significance in the evaluation of *Choose Life*) how to bridge gaps in knowledge between unsatisfactory proxy measures and the long-term goal of a programme. This latter problem can be illustrated by comparing a suicide prevention programme with a strategy to reduce coronary heart disease: whilst a three year heart disease programme might be deemed successful if it reduces levels of risk factors such as obesity or smoking, the links between self-harm incidence and suicide incidence are less clear-cut. Nonetheless, a Theories of Change approach has provided an evaluation framework that allowed researchers from a range of disciplines to work towards an integrated set of evaluation questions and helped to direct the team towards the selection of appropriate methods, including documentary analysis, interviews, group exercises and surveys [10]. An evaluation database was designed and utilised to store and retrieve data that were analysed iteratively and thematically. The data generated were, in addition, put in the context of international approaches to suicide prevention.

Whilst no separate economic data collection was undertaken, the research instruments developed for use in the study were deliberately informed by an economic perspective. Although imperfect, these allowed the evaluation team to consider the extent to which Choose Life stimulated additional local investment from their partnership organisations, including in-kind investments and the contribution of unpaid volunteers, targeted resources at relevant priority groups, spent their funding on proven effective practice and interventions, and stimulated local innovation.

In summary our approach aimed to provide a formative assessment of whether processes and short-term outcomes at far remove from the distal outcome – reduced rates of suicide – were being encouraged at a local and national level. It is not within the remit of this paper to discuss the findings of the evaluation – these are presented elsewhere [12], however, through the approach adopted the team were able to make recommendations concerning future investments in suicide prevention, sustainability, targeting of efforts, the integration of self-harm into suicide prevention strategies and the role of local and national partnerships. To illustrate we provide some specific examples. First, in relation to targeting local action we found that the long list of priority groups set by *Choose Life *was unhelpful since it led to rank ordering at a local level, and, in some cases, a lack of focus on local needs assessment. Second, in terms of sustainability, our data suggest that, whilst the initiative acted as an unprecedented rallying call for cross-sectoral planning to embed suicide prevention within the mental health improvement agenda, the local and national contexts made it difficult to bring together clinical and community approaches to prevention and treatment. Finally, in terms of defining the boundaries of *Choose Life *there was evidence of considerable variation across the programme of how 'high risk' suicidal behaviour was conceptualised and tackled. This suggests that nationally greater consideration needs to be given to how action on self-harm is integrated within a wider suicide prevention strategy.

## Summary

As discussed earlier, learning from the first phase of *Choose Life *was expected to feed into its second and more mature implementation phase. Many commentators who argue that public health and social care programmes, like medical interventions, should ideally be submitted to testing through randomised controlled trials acknowledge that this may be impossible in the early stages of implementation as national strategies struggle to find their local feet [36]. Instead they suggest that, once interventions have matured into stable entities, then they can be evaluated using gold standard approaches. Notwithstanding the impossibility of utilising a controlled approach to an intervention that has already been rolled out nationally, there are other difficulties with this suggestion. Above all, it is questionable whether social programmes ever become stable interventions – more likely they will strengthen or weaken and constantly evolve as part of a local and national system for tackling health improvement.

To help generate learning for the future we make the following recommendations that build on those contained in our final report [12]. First, an integrative framework such as Theories of Change should continue to shape purposive strategies for generating evidence. Second, work to determine nationally and locally acceptable proxy measures of success for phase two should be undertaken and resulting targets used to stimulate action rather than to create perverse incentives [37]. Third, continued efforts to learn about the effectiveness and cost-effectiveness of different strategies for intervening to promote mental health, prevent suicide, and alleviate its effects in different contexts should be prioritised – ongoing routine but comparable data collection on the uptake of different individual interventions and the resource contributions of unpaid volunteers to their delivery should be an important element of this process. Fourth, learning about the impact of different organisational structures on proxy indicators of progress should be undertaken through case-study evaluations of contrasting approaches. Fifth, whilst suicide prevention is a unique goal, efforts to capitalise on learning across policy domains about how to interpret/utilise evidence and mainstream good practice should be maximised. Finally, measuring trends in suicide rates will remain an important strand of monitoring progress only insofar as it is undertaken sensitively, with due cognisance of the small number of events involved and the distorting effect of 'natural' year on year fluctuations. With such a strategy in place, useful learning may emerge about how to impact positively on mental health and well-being and suicide prevention at strategic and local, as well as programme and practice, levels. Only then will we be in the position to judge whether we have appropriately measured the dog that doesn't bark in the night.

## Competing interests

The author(s) declare that they have no competing interests.

## Authors' contributions

All authors were involved in conception and design of the Choose Life evaluation or in data acquisition and interpretation. MM was the primary author of the paper with revisions provided by other authors, in particular, SP. All authors read and approved the final manuscript.

## Pre-publication history

The pre-publication history for this paper can be accessed here:


